# Postprandial lipaemia 10 and 34 hours after playing football: Does playing frequency affect the response?

**DOI:** 10.1371/journal.pone.0218043

**Published:** 2019-07-02

**Authors:** Darren J. Paul, George P. Nassis, Anissa C. Kerouani, Jens Bangsbo

**Affiliations:** 1 Aspetar – Qatar Orthopaedic and Sports Medicine Hospital, Exercise Sport Science Department, Doha, Qatar; 2 Department of Sports Science, City Unity College, Athens, Greece; 3 School of Physical Education and Sports Training, Shanghai University of Sport, Yangpu District, Shanghai, China; 4 Aspetar – Qatar Orthopaedic and Sports Medicine Hospital, Athletes Health and Performance Research Centre, Doha, Qatar; 5 Department of Nutrition, Exercise and Sports, Copenhagen Centre for Team Sport and Health, University of Copenhagen, Copenhagen, Denmark; University of Brasilia, BRAZIL

## Abstract

Elevated postprandial triglyceride (TG) is associated with increased risk of cardiovascular disease. The time window for the last bout beneficial effect on postprandial lipaemia after football play is unknown. The aim of the present study was to examine whether playing affects postprandial TG during 1.5 day of reduced activity. Eighteen males were randomly allocated to perform either 1 (1FOOT; n = 9; age = 33.0 ± 5.0 yrs; body mass index = 24.2 ± 3.6 kg/m^2^) or 3 (3FOOT) consecutive days of 60-min 5 vs 5 football (n = 9; age = 32.8 ± 5.2 yrs; body mass index = 26.2 ± 4.1 kg/m^2^) matches across a 5-day study period. They arrived to the laboratory 10 hrs and 34 hrs after the final football session and blood samples were collected at fasted (0 min) and 45, 90, 240 and 360 min post a high fat load meal. There were non significant increase for postprandial TG AUC (9.1%; p = 0.17; 95%CI = -0.43 to 2.0; ES = -0.23) and iAUC (14.2%; p = 0.43; 95%CI = -0.92 to 1.9; ES = -0.24) between 10 and 34 hrs after the 1FOOT. For the 3FOOT, there was a non significant decrease in postprandial TG AUC (-2.7%; p = 0.73; 95%CI = -2.0 to 1.5; ES = 0.05) and iAUC (-17.5%; p = 0.41; 95%ci = -2.5 to 1.1; ES = 0.31) from 10 to 34 hrs, respectively. Performing three consecutive days of football exercise may offer no greater protective effect for postprandial TG before a period of reduced activity, compared to a single session.

## Introduction

Sedentary behavior is an important issue in public health [1]. Prolonged time spent sitting or reclining (i.e., TV watching, computer-related activities, driving a car, etc.) is considered an important risk factor for abnormal lipid and glucose metabolism, type 2 diabetes, and all-cause mortality, independent of moderate-and-vigorous physical activity [1] and lower levels of physical activity [2,3]. The effect of sedentary behavior on lipid metabolism is worsened when it is combined with regular consumption of high energy dense foods [4]. The increased postprandial (post fed) triglyceride (TG) response following consumption of energy dense foods has also been linked to cardiovascular disease [4]. Though closely related, postprandial measures appear to be as useful as fasted TG for risk stratification [5]. Furthermore, postprandial TGs could be particularly useful for clinical trial setting where collecting fasting blood samples can become logistically complicated, thus, postprandial samples may improve patient compliance [6].

Several different models of sedentary behaviour exist, including training cessation for athletes, increasing sitting time, reducing daily ambulatory activity and bed rest. All of these likely induce negative physiological effects that may provide information relating to metabolic dysfunction. The negative effect of low activity levels on postprandial TG have been reported in some studies [7,8,9,10]. Postprandial TG area under curve (AUC) has shown to increase when the step count is reduced from an average ~10,500 to ~1,150 steps per day over a two-week period in a group of young healthy men [4]. In trained athletes, postprandial TG concentrations has shown to increase by over one third within 60h after stopping training [8]. Zhang et al., [9] reported exercise 12 hrs prior to a high fat meal significantly reduced postprandial TG in men with hypertriglyceridemia, whereas exercising 24 hrs prior to the meal does not. Pafili et al. [10] reported postprandial TG AUC was lower 16 hrs after exercise (day 1), compared with control, whereas no difference existed between control and 40 hrs after exercise (day 2). Such evidence suggests a possible time course response regarding the effects of exercise on postprandial TG, although this may be dependent on the exercise modality.

Several studies have shown endurance, resistance and high intensity exercise to independently lower postprandial TG [11–16]. However, these may not be the chosen exercise habits of many individuals worldwide. Only recently have the benefits of playing football on postprandial TG been examined. The findings of these studies have shown that playing football reduces postprandial TG in overweight individuals [17], and adolescents [18] and may reduce postprandial TG similarly to continuous running, but with lower rating of perceived exertion [13]. Previous work has also shown some health benefits are maintained when reducing football training from three to one session per week, though these were fasted and not postprandial measures [19]. Such findings may have important clinical implications, particularly as football has worldwide popularity and may be particularly appealing for hard to reach populations [20]. Therefore, the aim of the present study was to examine whether different frequency of playing football (1 session vs 3 sessions) affects postprandial TG during 1.5 day of reduced activity.

## Materials and methods

### Participants

Eighteen males were randomly allocated to perform either 1 (1FOOT, day 3; n = 9; age = 33.0 ± 5.0 yrs; body mass index = 24.2 ± 3.6 kg/m^2^) or 3 football (3FOOT, day 1, 2 and 3; n = 9; age = 32.8 ± 5.2 yrs; body mass index = 26.2 ± 4.1 kg/m^2^) sessions across a 5-day study period. Participants arrived to the laboratory 10 hrs (day 4) and 34 hrs (day 5) after the final football session and blood samples were collected at fasted (0 min) and 45, 120, 240 and 360 min post a high fat load meal. The study was approved by the Qatar Anti Doping Laboratory ethics committee (number F2015000112), and was in accordance with the Declaration of Helsinki. Written informed consent was obtained from all study participants prior to commencing.

General exclusion criteria were: (1) cardiovascular, pulmonary or metabolic disease, (2) absolute contraindications to exercise testing as established by the American College of Sports Medicine, (3) dietary restrictions regarding the meals provided. In order to control for confounding variables, participants were instructed to (1) fasting was standardized for 10 and 34 hrs before the fat meal, (2) abstain from caffeine, tobacco, and vitamin supplements for 24 hrs before blood sampling, and (3) awake between 06:00 and 07:00 hrs prior to laboratory testing. Participants were asked to refrain from any other physical activity, besides that of the exercise intervention, for the 36 hrs before the start of pre intervention measures.

### Preliminary measures

Body mass and stature were recorded using a combined scale and stadiometer (SECA 769, Fremont, CA, US) with body mass index calculated. Waist and hip circumferences were measured at visual narrowing of the waist between the iliocristale and 10^th^ rib landmarks (waist) and the girth of the buttocks at the level of the posterior protuberance. Body fat was estimated using the InBody Bioelectrical Impedance Analysis Systems (Inbody 720, Biospace, Seoul, Korea).

All participants performed the Yo Yo intermittent endurance test level 1 (YYIETL1), in an air-conditioned sports hall on artificial turf to establish maximal heart rate (HR) and cardiorespiratory fitness. The YYIETL1 test consists of 2 x 20 m shuttle runs performed at increasing running speeds, interspersed with 5 s of active recovery, during which the participants jogged around a cone placed 2.5 m behind the start/finishing line. The speeds were controlled by audio and the test was terminated when the participant was no longer able to maintain the required speed for 2 consecutive runs. The total distance (metres) covered represented the test result.

### Experimental procedure

**Fig 1:**
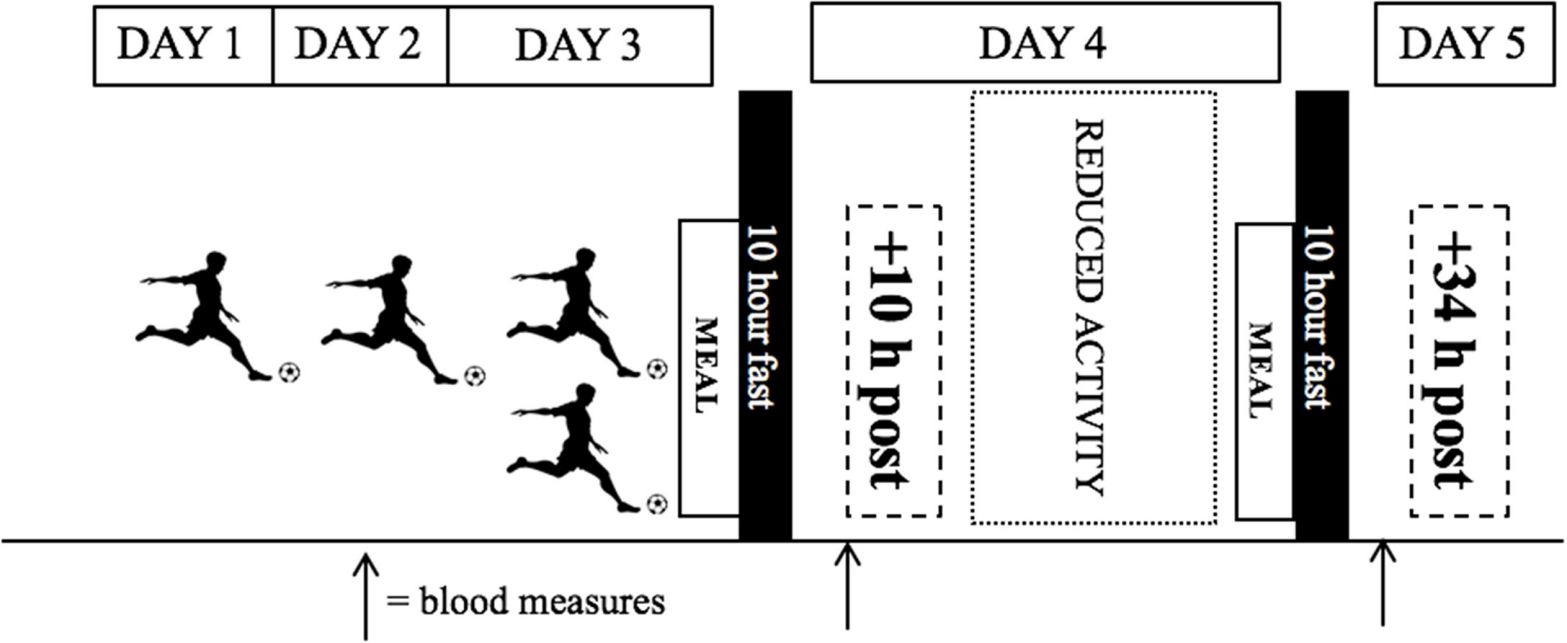
Schematic representation of study design.

On day 1 participants arrived to the laboratory at approximately 0800 hrs after an overnight fast (10 hrs) (Fig 1). Following a rest period in a seated position and debrief of the experiment, a cannula was inserted into an antecubital vein and a baseline venous sample was collected by a qualified phlebotomist. Participants were given the high fat load meal (798 kcal; 59g fat; 55 g carbohydrates), in the form of a Belgian chocolate ice cream (Haagen Daas) and whipping cream (Elmlea) drink. The meal quantity was adjusted relative to body mass, comprised of 1.2 g fat/kg, 1.2 g carbohydrate/kg and 0.4g protein/kg, being 17 ml of cream per 100 ml ice cream. The meal was well tolerated by all participants. A stopwatch was started when participants began consuming and the time taken to complete was recorded (mean time taken = 6.3 ± 1.5 mins) and replicated in the subsequent trial. The stopwatch was reset and time taken for further blood samples again at 45, 120, 240 and 360 min post meal consumption. Water was available ad libitum during the first trial; the volume ingested was recorded and replicated in the subsequent trial. Participants rested (reading, working quietly, watching television) throughout each observation period.

Participants were instructed to wear an Actigraph activity monitor (Actigraph, wGT3X-BT, Actigraph, Florida, USA) across the 5-day experimental period to monitor step counts, this included the three days of the intervention (3FOOT vs 1FOOT and 2 days of habitual activity) as well as the two days of trials (10 and 34 hrs). The activity monitor was placed on the hip as this position has shown to be the most reliable for estimating energy expenditure [21]. Participants were instructed to put the device on as soon as they woke up and take off only to shower and sleep. Estimated energy expenditure was calculated from the integrated tri axial accelerometer using the Freedson vector magnitude equation [22].

All sessions for both groups consisted of 60 min (2 x 30 min) small sided game (5 vs 5) performed on an artificial outdoor court (36.5 x 27.5 m). All matches were played at the same time of day (20:00 hrs) and each player in turn played as goalkeeper for the matches. The activity profiles during the football match were recorded using the same Actigraph activity monitor and session rating of perceived exertion (sRPE) was recorded within 5 min of exercise completion using the CR-10 Borg scale and player load calculated (sRPE x minutes) [23]. Perceived recovery status (PRS) was assessed on a scale of 0 (very poorly recovered/extremely tired) to 10 (very well recovered/highly energetic) approximately 10mins prior to each session [24].

Following the last football session and the evening prior to the first visit to the laboratory, all participants consumed a standardised meal (12-inch Subway meat ball marinara sandwich) providing 960 kcal; 36 g fat, 120 g carbohydrates and 42 g protein. Participants were instructed to consume the sandwich, and nothing else, after the last football session. Participants consumed the same meal (Subway sandwich) the evening of trial one (day 1) to ensure the dietary intake was replicated prior to the second trial (day 2) experiment.

### Analytical procedures

Blood samples were collected into 9-ml potassium-EDTA Monovettes (Sarstedt, Leicester, UK) or serum separate tubes (SST)-gel/clot activator. Serum was obtained by centrifugation (15 min at 1500*g* and at room temperature) and was collected and stored at -80 ^o^C for further analysis. Analyses of all samples from the same participant were done in a single batch. The spectrophotometric analyses were performed on an automated clinical chemistry system (Dimension Clinical Chemistry System—Xpand) to determine the level of TG, insulin, glucose and non-esterified fatty acids (Abbott-Cell Dyn 3700).

### Calculations

Triglyceride, glucose and insulin responses were assessed as the AUC and incremental areas under the concentration-versus-time curves (iAUC). Incremental area under the curve was calculated using the trapezoid rule and calculated by subtracting, from the postprandial area, the mean baseline value extrapolated over 6 hrs; thus they reflect changes occurring after the meal. Whole-body insulin sensitivity was assessed with the homeostasis model assessment (HOMA) of insulin resistance index from serum fasting data as: insulin (μU/ml) × glucose (mmol/l)/22.5) [25] and with the Matsuda’s composite whole body insulin sensitivity index (ISI) in the postprandial state as: 10,000/square root of (fasting glucose (mg/dl) × fasting insulin (μU/ml) × mean postprandial glucose (mg/dl) × mean postprandial insulin (μU/ml)) [26].

### Statistical analyses

Data were tested for normality with the Kolmogorov-Smirnov test, with all data shown to be normally distributed. A 2x2x5 (group x hours after the match x blood sampling time points) analysis of variance (ANOVA) was used for all comparisons between the two trials. When significant overall differences between the trials was found, a Bonferonni post hoc test was applied. Students’ unpaired t-test was used to compare baseline characteristics between 1FOOT and 3FOOT groups. Statistical significance was set at p< 0.05 and 95% confidence intervals (CI) and Cohen’s d effect sizes (ES) were calculated. The criteria to interpret the magnitude of the ES were: > 0.2: small, > 0.6: moderate, > 1.2: large and >2.0: very large) [27]. All statistical analyses were performed using the Statistical Package for the Social Sciences (SPSS), version 21.0. Descriptive statistics of the data are presented as means ± standard deviation (SD) unless otherwise stated.

## Results

### Match load response

Estimated energy expenditure was greater for day two football session (408.7 ± 68.8 kcals) than day one (364.4 ± 40.6 kcals; p< 0.01; 95%CI = 74.17 to 14.42; ES = 0.78) and day three (342.7 ± 87.9 kcals; p< 0.01; 95%CI = 36.12 to 95.88; ES = 0.83) in the 3FOOT group. No other difference was found between the 3FOOT consecutive days of football session for any of the metrics relating to time in different zones for energy expenditure. In the 3FOOT condition we found no difference for sRPE (day one: 354 ± 66.0, day two: 336 ± 102, and day three: 282 ± 56), although a large effect size was calculated for day one vs day three; ES = 1.17. There were no differences for PRS (day one: 6.9 ± 1.5, day two: 6.6 ± 1.5, day three: 5.8 ± 1.6) between the 3 days for the 3FOOT group. We found no differences for sRPE between 1FOOT (282 ± 56) and 3FOOT (313 ± 16) (ES = 0.75) for the only (1FOOT) and day 3 of football matches (3FOOT). There were no differences between groups for the number of steps for the three days prior to the first laboratory visit and there was no difference for the number of steps between 1FOOT (4897 ± 1504 steps/day) and 3FOOT (4753 ± 2071 steps/day) during day 4 of the experiment (10 hrs), inferring that participants performed a similar activity level prior to day 5 of the experiment (34 hrs).

### Metabolic response

**Fig 2:**
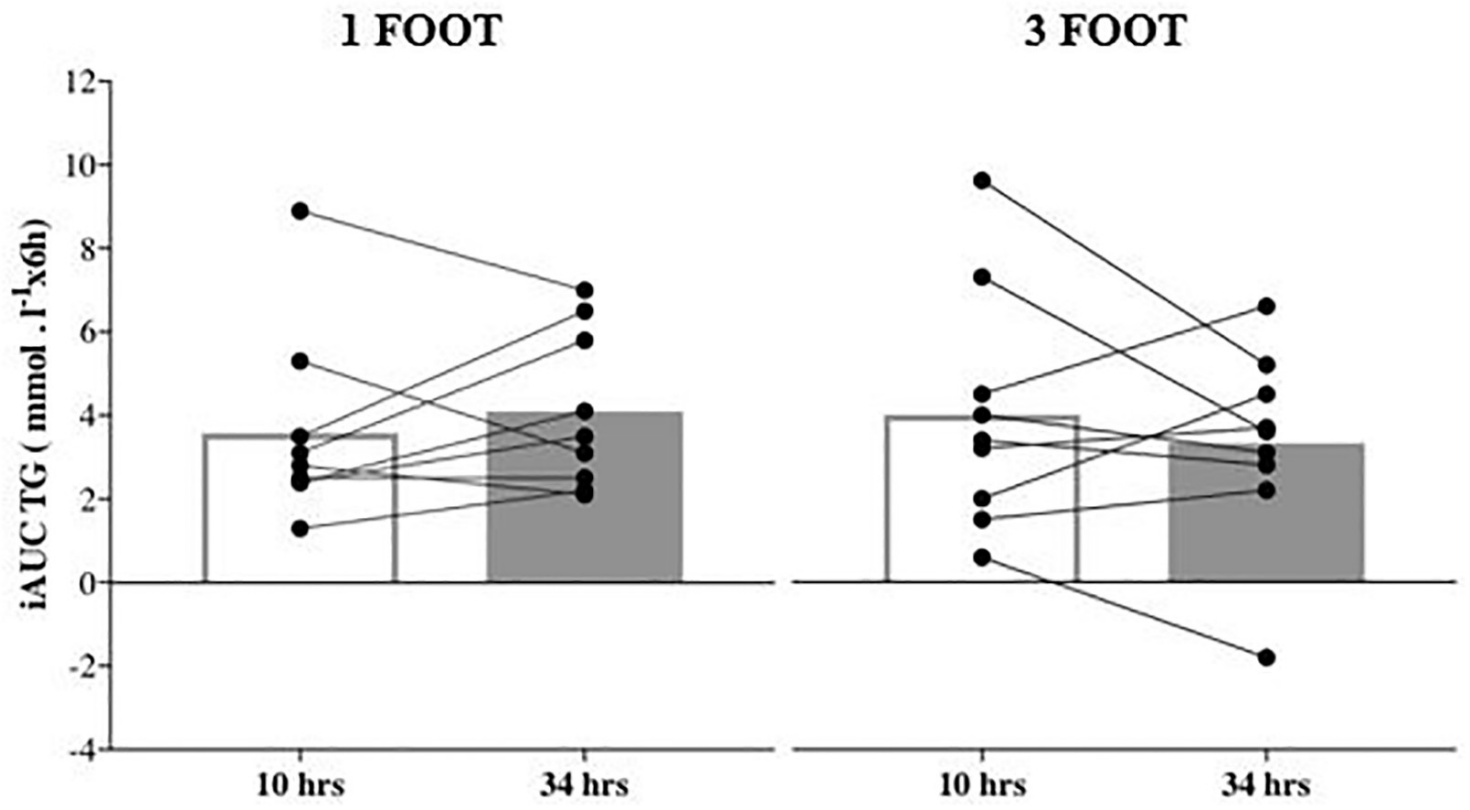
Average value and individual responses for the postprandial triglyceride incremental area under the curve (iAUC) response to a high fat meal at 10 hrs and 34 hrs after the last football sessions. (1 FOOT: One 60-min football session; 3 FOOT: Three consecutive 60min football sessions).

There was a non-significant increase in postprandial TG AUC (9.1%; p = 0.17; 95%CI = -0.43 to 2.0; ES = -0.23) and iAUC (14.2%; p = 0.43; 95%CI = -0.92 to 1.9; ES = -0.24) (Fig 2) between 10 and 34 hrs after the 1FOOT. For the 3FOOT, there was a non-significant decrease in postprandial TG AUC (-2.7%; p = 0.73; 95%CI = -2.0 to 1.5; ES = 0.05) and iAUC (-17.5%; p = 0.41; 95%ci = -2.5 to 1.1; ES = 0.31) from 10 to 34 hrs, respectively (Table 1).

**Table 1:** Baseline blood lipids concentration (fasted), area under the curve (AUC) and incremental area under the curve (iAUC) for both the 1FOOT and 3FOOT at +10 and +34 h after the last football session (data are presented as mean ± SD).

Measure	Group	10 hrs	34 hrs	10 to 34 hrs % change	Statistical analysis
TG AUC (mmol.l^-1^ 6h^-1^)	1FOOT	8.6 (3.87)	9.4 (3.05)	9.1	p = 0.17; 95%CI = -0.43 to 2.01; ES = -0.23
	3FOOT	9.5 (4.55)	9.2 (3.49)	-2.7	p = 0.73; 95%CI = -2.03 to 1.50; ES = 0.07
Insulin AUC (mmol.l^-1^ 6h^-1^)	1FOOT	51.9 (27.99)	58.7 (16.52)	13.1	p = 0.28; 95%CI = -6.98 to 20.63; ES = 0.12
	3FOOT	43.9 (22.48)	66.1 (26.97)	50.4	p = 0.05; 95%CI = -0.12 to 44.48; ES = -0.89
Insulin iAUC (mmol.l^-1^ 6h^-1^)	1FOOT	28.0 (21.56)	32.4 (9.94)	15.8	p = 0.39; 95%CI = -6.89 to 15.4; ES = -0.26
	3FOOT	17.8 (16.01)	37.0 (19.03)	108.1	p = 0.02; 95%CI = 2.77 to 35.73; ES = -1.09
Glucose AUC (mmol.l^-1^ 6h^-1^)	1FOOT	28.2 (2.11)	30.1 (1.93)	6.8	p = 0.06; 95%CI = -0.12 to 3.99; ES = -0.93
	3FOOT	28.6 (2.94)	30.2 (3.00)	5.5	p = 0.01; 95%CI = 0.33 to 2.79; ES = -0.52
Glucose iAUC (mmol.l^-1^ 6h^-1^)	1FOOT	-2.9 (2.26)	-1.3 (1.99)	-54.8	p = 0.16; 95%CI = -0.82 to 4.05; ES = -0.95
	3FOOT	-2.0 (1.28)	-0.7 (1.22)	-65.2	p = 0.03; 95%CI = 0.12 to 2.53; ES = -1.06
NEFA AUC (mmol.l^-1^ 6h^-1^)	1FOOT	3.8 (0.38)	3.7 (0.45)	3.7	p = 0.29; 95%CI = -0.41 to 0.14; ES = 0.24
	3FOOT	4.6 (1.31)	3.9 (0.65)	-15.6	p = 0.04; 95%CI = -1.42 to -0.02; ES = 0.69
NEFA iAUC (mmol.l^-1^ 6h^-1^)	1FOOT	0.2 (0.53)	0.1 (0.37)	-63.2	p = 0.52; 95%CI = -0.55 to 0.30; ES = 0.21
	3FOOT	0.3 (0.46)	-0.3 (0.71)	-63.2	p = 0.08; 95%CI = -1.26 to 0.09; ES = 1.00
HOMA	1FOOT	0.9 (0.33)	1.0 (0.29)	11.0	p = 0.40; 95%CI = -0.16 to 0.36; ES = -0.32
	3FOOT	1.0 (0.34)	1.1 (0.46)	12.4	p = 0.44; 95%CI = -0.22 to 0.45; ES = -0.24
ISI Matsuda	1FOOT	19.1 (11.47)	13.4 (3.79)	-29.9	p = 0.07; 95%CI = -12.2 to 0.77; ES = 0.66
	3FOOT	18.9 (8.23)	12.7 (5.33)	-31.9	p = 0.03; 95%CI = -11.39 to -0.65; ES = 0.86

TG = Triglyceride, NEFA = Non esterified fatty acids, HOMA = Homeostasis model of assessment; ISI = Insulinomic Index

**Fig 3:**
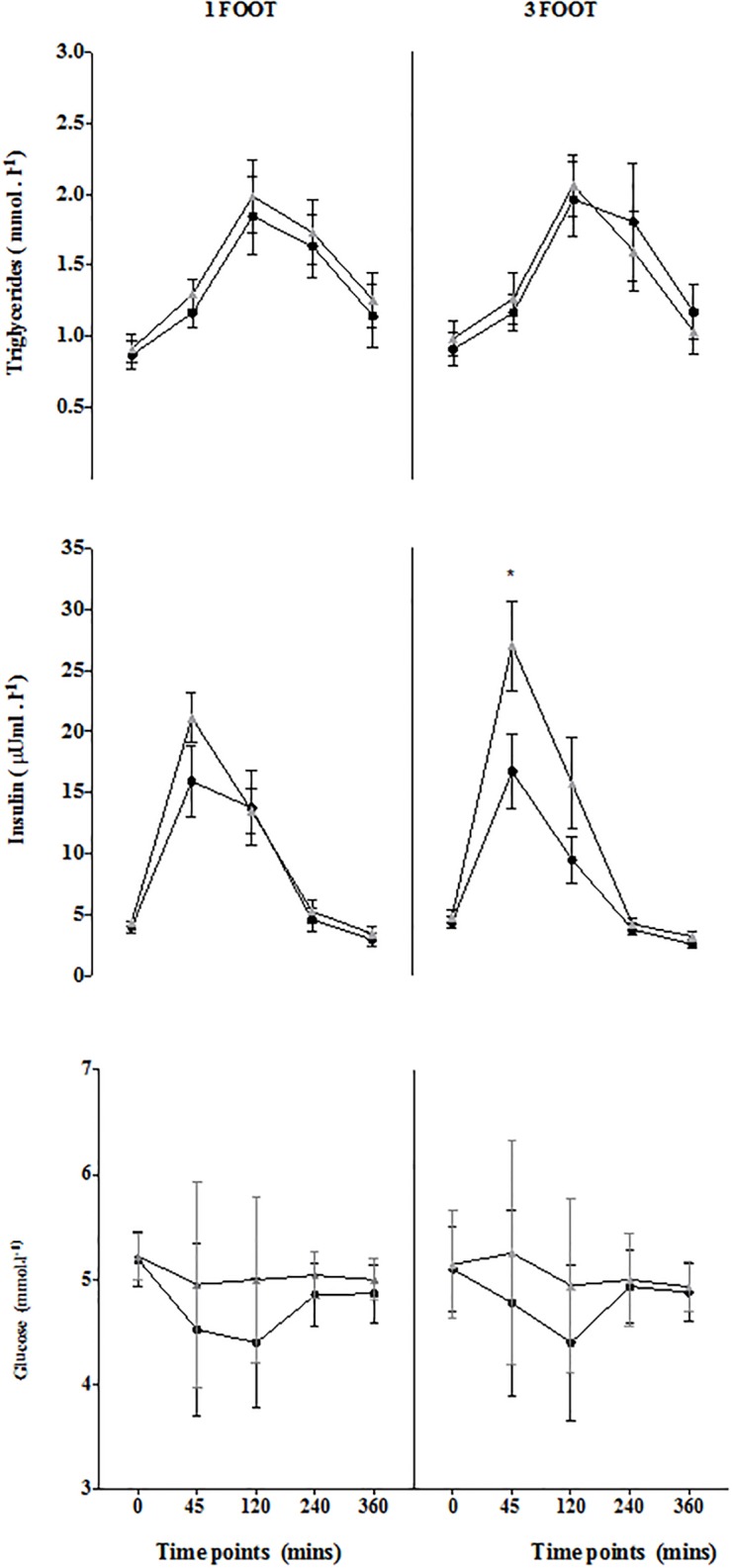
Fasting and postprandial time course from the high fat meal after one (left column) and three consecutive (right column) 60 min football sessions. Values are means+/-SE. Black circles represent 10 hrs and grey triangles represent 34 hrs after the sessions. *p< 0.01 between conditions.

Insulin iAUC increased from 34 to 10 hrs (Table 1) in the 3FOOT and this was due to the higher peak at 45 mins (p< 0.05, 95%CI = 19.84 to 0.80; ES = 0.5, Fig 3). Postprandial glucose iAUC was greater at 34 hrs compared with 10 hrs (p = 0.03; 95%CI = 0.12 to 2.53; ES = -1.06, Table 1) for the 3FOOT. The postprandial NEFA AUC were shown to be lower from the 10 to 34 hrs time points (Table 1).

## Discussion

The aim of the present study was to examine whether different frequency of playing football (1 session vs 3 sessions) affects postprandial TG during 1.5 day of reduced activity. We found no difference for postprandial TG incremental area under the curve from 10 to 34 hrs following either one single or three consecutive 60 min sessions of playing football. Our findings have important clinical implications, notably that there does not appear to be an additive effect from three consecutive days of playing football in the evening 10 and 34 hrs post the last bout of exercise, compared to solely one football session.

In comparison to our study, Zhang et al., [9] reported exercising 12 hrs prior to a high fat meal significantly reduced postprandial TG in men with hypertriglyceridemia, whereas exercising 24 hrs prior to the meal does not. In the study by Pafili et al, [10] postprandial TG area under the curve was 12% lower on day one (10 hrs) compared to control and returned to baseline 40 hrs post (day 2), while there were no differences for postprandial TG incremental area under the curve between trials. In trained individuals, the benefits of a single acute bout of exercise on postprandial TG seem to be lost between 15 and 60 hrs [28].

The fact we showed no difference between the 10 and 34 hrs may be explained by several reasons. While we did not perform pre intervention measure of postprandial TG, several studies, including previous work from ourselves, have shown the lowering effects of exercise from control to post training intervention. Energy expenditure and lipoprotein lipase (LpL) are suggested to be important factors for postprandial TG reduction, however, evidence has shown a dissociation of energy expenditure from the magnitude of postprandial TG attenuation [11]. While the energy expenditure in our study is lower than the recommended amount required for lowering postprandial lipaemia (450–600 kcal) [29], the intense muscle contractions associated with playing football (eccentric muscle contractions) may have an effect through a mechanism that seems to be less related to energy expenditure [10]. Since eccentric exercise may be a potent factor for lowering postprandial TG, there may be suggestions that this maintains any improvements. Lipoprotein lipase has shown to be effected by highly demanding activity. Part of the rationale for LpL being an important factor in lowering postprandial TG is that they follow a similar time course of action [10,11]. However, it seems that when TG remains stable, LpL activity may not be modified [30].

The light ambulatory contractions of habitual daily activity performed during the 10 and 34 hrs time period may have been enough to maintain lower levels of postprandial TG, since LpL has shown to be sensitive to small changes in ambulatory daily activity, albeit this was reported in rats [31]. While the participants were instructed to largely reduce activity level, the number of steps performed (~4800) may have been enough to avoid large excursions in postprandial TG. For instance, reductions in postprandial TG have been reported following walking, but not standing, in a group of healthy normolipidaemic Japanese men [15], thus, demonstrating its sensitivity to low levels of activity. Furthermore, research has shown as low as 2 min of walking at 60% of VO_2_max every 30 min, improved postprandial TG area under the curve [32]. Thus, sustaining a low level of habitual ambulatory activity seems to be enough to hold off unfavorable effects of reduced activity on postprandial TG and may have been the case in our study.

It is important to consider the participant characteristics, as well as the synergistic interaction between previous training experience and acute exercise, may be a factor for the results in our study. Though our participants were not highly trained individuals, they had previous experience playing football. Research has shown that trained individuals may have a low chylomicron-TG half-life which may partly be due to a reduction in the fasting TG pool size and partly to a direct effect of chronic exercise on the TG removal system [33]. It is also reported that the adverse effects of low activity levels may only occur when there is a large contrast between training and non training periods of exercise and low level activity (i.e. bed rest studies) and that it is sustained over a period of consecutive days (e.g six days) [4].

The 3FOOT group showed a substantial increase (~108%) in insulin incremental area under curve from 10 to 34 hrs, compared to the 1FOOT group (~ 16%). This change in the 3FOOT is probably a manifestation of the 40% lower concentrations at 45 min on day 4 (10 hrs). The negative effect of low levels of activity on insulin response have been shown after only a few days 3. Pafili et al [10] showed insulin response to be similar between day 1 and pre measures, a transient increase in insulin concentration was observed 2 hrs after the consumption of the meal on day 2, resulting in an increase of the postprandial insulin iAUC. We can only speculate, but the higher insulin concentrations may be the result of greater secretion from the pancreatic cells as well as decreased insulin clearance and reduced binding to insulin receptors and/or clearance by the liver [34]. This may be to compensate for the decrease in skeletal muscle insulin signalling, at least during the early stages of reduced activity levels in an attempt to conserve euglycemia [35]. The alteration may be the result of decreased insulin action accompanied by decreased GLUT-4 protein, which is important in the regulation of insulin stimulated glucose uptake by the tissues [36]. However, these remain speculative and a better understanding of the time course of postprandial insulin response following exercise and reduced activity is required.

The different changes in insulin from day 4 to 5 seen between 1FOOT and 3FOOT may be a combination of a decreased concentration on day 4 (the day after the last football match), but also a large increase on day 5 (after the period of inactivity). We speculate a greater muscle damage in 3FOOT which may have been detected 34-hour post. Paschalis et al., [37] demonstrated increased glucose and insulin levels and as a result increased levels of HOMA after a first bout of eccentric exercise. On the contrary, these adverse effects of acute eccentric exercise on insulin resistance subsided after chronic exercise, showing decreased resting levels of insulin, glucose and HOMA. Paschalis et al., [37] also showed acute exercise significantly modified levels of TG, as well as total, high and low density lipoprotein cholesterol only after eccentric exercise at week 1 and not week 8.

Our results may also be explained by the composition of the high fat meal consumed. Chong et al., [38] demonstrated that a high fat meal with increased fructose potentiates postprandial lipaemia in the acute setting. They also reported a significantly higher rise in very low density TG (Sf 20–400 lipoprotein-TG) concentration and a later peak in chylomicron-TG (Sf—400 lipoprotein-TG) concentration after fructose and were consistent with a delay in clearance or absorption. The fructose produced a significantly smaller glycemic excursion and induced a significantly smaller increase in insulin concentration than did glucose and may explain the increase in insulin levels. However, we have previously shown increases in postprandial using this same meal and therefore it may be more related to the exercise characteristics.

## Conclusion

In conclusion there was no difference for postprandial TG incremental area under the curve from 10 to 34 hrs following either one single, or three consecutive days of playing 60 minutes of football. Performing three consecutive days of football exercise may offer no greater protective effect for postprandial TG before a period of reduced activity, compared to a single session. Two-hour insulin response increased from 10 to 34 hrs, suggesting impaired sensitivity.
